# Interface Engineering for Perovskite Solar Cells Based on 2D-Materials: A Physics Point of View

**DOI:** 10.3390/ma14195843

**Published:** 2021-10-06

**Authors:** Rosaria Verduci, Antonio Agresti, Valentino Romano, Giovanna D’Angelo

**Affiliations:** 1 Department of ChiBioFarAm, University of Messina, 98166 Messina, Italy; rosaria.verduci@unime.it; 2 C.H.O.S.E. (Centre for Hybrid and Organic Solar Energy), Department of Electronic Engineering, University of Rome Tor Vergata, 00133 Rome, Italy; 3 Department of Mathematical and Computer Science, Physical Sciences and Earth Sciences (MIFT), University of Messina, 98166 Messina, Italy; giovanna.dangelo@unime.it

**Keywords:** perovskite solar cells, 2D materials, interface engineering, hot carriers, additives, crystallization, work function tuning

## Abstract

The last decade has witnessed the advance of metal halide perovskites as a promising low-cost and efficient class of light harvesters used in solar cells (SCs). Remarkably, the efficiency of lab-scale perovskite solar cells (PSCs) reached a power conversion efficiency of 25.5% in just ~10 years of research, rivalling the current record of 26.1% for Si-based PVs. To further boost the performances of PSCs, the use of 2D materials (such as graphene, transition metal dichalcogenides and transition metal carbides, nitrides and carbonitrides) has been proposed, thanks to their remarkable optoelectronic properties (that can be tuned with proper chemical composition engineering) and chemical stability. In particular, 2D materials have been demonstrated as promising candidates for (i) accelerating hot carrier transfer across the interfaces between the perovskite and the charge extraction layers; (ii) improving the crystallization of the perovskite layers (when used as additives in the precursor solution); (iii) favoring electronic bands alignment through tuning of the work function. In this mini-review, we discuss the physical mechanisms underlying the increased efficiency of 2D material-based PSCs, focusing on the three aforementioned effects.

## 1. Introduction

In the last few decades, concerns about the depletion of fossil fuels, environmental pollution and climate changes have encouraged the transition to an economy based on sustainable and renewable sources. Among these, sunlight, with a potential of 23,000 TWy/y (y = year), is the main environmental friendly source capable of meeting the current (~17.6 TWy for 2020) [1] and future (27 TWy by 2050) energy demand [2]. Nowadays, photovoltaic (PV) systems represent the most promising and mature technological solution to harness and convert solar energy into electricity. In this context, metal halide perovskites emerged as promising and outstanding PV light-harvesters for the fabrication of solar cells (SCs) because of panchromatic absorption of the visible spectrum (absorption coefficient >10^5^ cm^−1^) [3,4], high charge carrier diffusion lengths (in the µm range) [3], good charge carrier mobility (24–105 cm^2^ V^−1^s^−1^ for MAPbI_3_) [3,5] and the possibility to realise thin films with low cost processing of solution precursors [6,7]. The rapid development of perovskite-based materials has its roots in the intensive research performed at the beginning of 90′s, when researchers at IBM T.J. Watson Research Center (Yorktown Heights, NY, USA) explored the optoelectronic properties of halide perovskites for commercial applications in light-emitting devices [8]. After a long silence, these materials came back on to the scene in 2009, when Kojima et al. prepared methyl ammonium lead halide (MAPbX_3_; where MA = CH_3_NH_3_^+^, X = halide anion) perovskites and investigated their optoelectronic properties [9]. Moreover, the same authors fabricated dye-sensitized SCs using MAPbX_3_ visible light sensitizer demonstrating good power conversion efficiency (PCE) of ~3.8%. The first report of a solid-state perovskite-based SC (PSC) appeared in 2012 with a PCE of 9.7% and 500 h stability [10]. Afterwards, the performances of PSCs increased exponentially reaching PCE of 25.5% in ~10 research years [11]. This represents a remarkable result, considering that comparable performances are shown by the well-established Si and GaAs technologies (current records of 26.1% and 27.8%, respectively) [11]. Thus, PSCs are a disruptive technology in photovoltaics due to their superb performance and potentially cheap fabrication that has pushed the PV scientific community in developing stable perovskite ink, suitable for low-cost printing techniques [6]. As the matter of fact, understanding of precursor solution chemistry is helpful to prepare high-quality perovskite films and eventually high-efficiency devices. Moreover, even if organic–inorganic perovskites are known to be defect tolerant, defect engineering at interfaces and in the bulk is important for hysteresis-free, stable and high-efficiency PSCs. In addition, a PSC is usually realized by employing a sandwiched structure composed of several components: a transparent conductive electrode (such as fluorine tin oxide—FTO or indium tin oxide—ITO), an electron transporting layer (ETL), a perovskite absorbing layer, a hole-transporting layer (HTL) and a metal contact (such as Ag, Au) [6,12]. The engineering of such a complex structure requires the development of a comprehensive knowledge of chemistry, electronic engineering, material science and physics leading to an astonishing increase in publications per year in the last decade (from 21 documents in 2012 to about 4000 in 2020, Scopus sources). Depending on the order in which the ETL and the HTL are arranged, the architecture of a PSC can be categorized as direct (n-i-p) or inverted (p-i-n) [13]. In the n-i-p configuration, titanium dioxide (TiO_2_) and tin dioxide (SnO_2_) are the most used ETL while 2,2(7,7)-tetrakis-(N,N-dipmethoxyphenylamine)9,9(-spirobifluorene)—spiro-OMeTAD—and poly[bis(4-phenil) (2,4,6,-trimethylphenyl) amine] (PTAA) represent the most common HTLs [4,12]. In the inverted p-i-n PSC, PTAA is commonly used as HTL while [6,6]-phenyl-c61-butyric acid methyl ester (PCBM) and C_60_ fullerene are the typical ETLs [4,12]. Furthermore, depending on the structure of the ETL, PSCs can be further classified into planar and mesoporous [13]. Specifically, the mesoporous configuration differs from the planar one for the presence of a mesoporous metal oxide layer (usually m-TiO_2_) between the compact hole-blocking layer (usually a compact TiO_2_ –cTiO_2_- realized by a high temperature spry pyrolysis process) or HTL and the perovskite layer [4,12].

In SCs, further PCE improvements can be realised through an optimal charge carrier transport and collection, an improved crystal growth of the layers and an optimal energy-level alignment at the interfaces. Within this context, the use of two-dimensional (2D) materials has been demonstrated as a successful strategy because of their peculiar opto-electronic properties arising from quantum-confinement effects [14]. In 2004, Geim and Novoselov isolated experimentally for the first time the forefather of 2D materials, graphene. It consists of a single layer of C atoms arranged in a honey-comb structure, characterised by a semi-metallic electronic behaviour (i.e., its band gap is 0, Table 1) and an electronic dispersion curve responsible for a relativistic-like transport of charge carriers (resulting in high charge carrier mobilities, Table 1) [15,16].

In principle, all layered crystals (i.e., characterised by strong intra-layer covalent bonds and weak inter-layer van der Waals interactions) can be easily cleaved in the corresponding 2D form [28]. Thus, through the years several 2D materials have been discovered and characterised. Among them, transition metal dichalcogenides (TMDCs) and transition metal carbides, nitrides and carbonitrides (all known as MXenes) are at the forefront of current research efforts because of their chemical stability, good charge carrier mobility (Table 1) and the possibility to tune their band gaps by varying their chemical composition [29,30]. Furthermore, several techniques are being exploited and improved for the production of high quality 2D materials, paving the way for their large scale production. Some examples are chemical vapor deposition (CVD), growth on SiC, molecular beam epitaxy, mechanical and liquid phase exfoliation, etc. [31,32].

In this work, we will present some representative results concerning the use of 2D materials in PSCs. Since this is a very hot topic in the current research trends, we do not aim to discuss the literature results (which has been reviewed by several interesting papers) in depth [33,34,35,36], but to highlight some physical mechanisms behind the successful implementation of 2D materials within the PSCs structure. In particular, the discussion will cover the effects on: (i) hot-carriers; (ii) crystallization of the perovskite layer and (iii) tuning of energy levels. Thus, our work will give a first idea of the promises and advantages in synergistically using such technologies for improved PV performance.

## 2. The Family of 2D Materials

**Figure 1:**
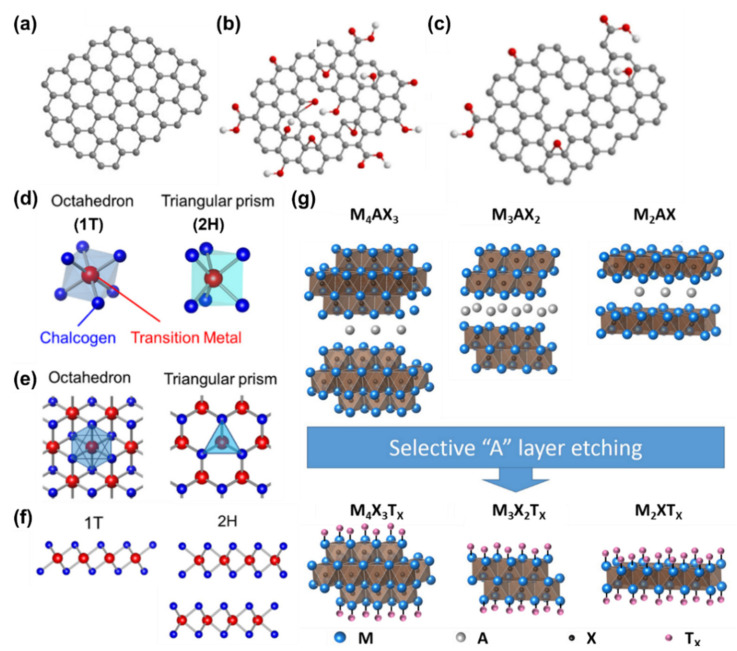
Schematic representation of: (**a**) graphene, (**b**) graphene oxide and (**c**) reduced graphene oxide. Figures adapted from ref. [37] Copyright Elsevier Ltd.@2020. (**d**) Octahedral (1T) and triangular (2H) prismatic structures of transition metal dichalcogenides (TMDCs). (**e**) Top and (**f**) side view of 1T and 2H structures. Figures adapted from ref. [38] Copyright IOP Publishing Ltd.@2015. (**g**) Schematic illustration of the production of MXenes from M_n+1_AX_n_ phases. Taken and adapted from ref. [39]. Copyright Wikipedia @2021.

Graphene, the first 2D material isolated in 2004 by Geim and Novoselov, consists of a single layer of sp^2^ hybridized C atoms arranged in a honeycomb crystal lattice (Figure 1a). 

The quantum nature of 2D materials leads to peculiar physico-chemical properties. For example, graphene is a zero-bandgap semi-metal (its valence and conduction bands meet at the Dirac point) with an ambipolar electrical transport behaviour showing charge carrier mobilities as high as 2∙10^4^ cm^2^ V^−1^ s^−1^. A graphene single layer exhibits a high transparency (97.7% in the visible range) [40,41] resulting in a promising material for the replacement of transparent conductive materials such as ITO and FTO. In addition, graphene has an elastic limit of ~20% and a weight of only 0.77 mg m^−2^, making it a suitable material for the realisation of flexible and light devices [42,43]. Finally, it is worth mentioning that graphene’s properties can be changed by chemical functionalization with oxygen-containing groups. For example, pristine graphene is hydrophobic (thus it cannot be well dispersed in water) [44], however the oxidation of graphene to graphene oxide (GO) permits uniform dispersion in aqueous solutions thanks to the introduction of many functional hydrophilic groups (like epoxide and hydroxyl groups on the planar surface and carboxylic groups at the edges) [45]. However, because of the distortion of the sp^2^ conjugation of the hexagonal graphene lattice induced by the presence of covalent C–O bonds, GO is an insulator [46]. Nevertheless, after chemical reduction processes GO is transformed into reduced graphene oxide (rGO) that is characterized by high conductivity and transparency resulting in an attractive and promising material for optoelectronic applications [47]. Figure 1b,c schematically represents the crystal structure of GO and rGO, respectively. The TMDCs are a large family of 2D materials with chemical formula MX_2_, where M is a transition metal atom (such as Mo, W, Ta, Nb, etc.) and X is a chalcogen atom (for example S, Se or Te) [48]. Specifically, a TMDC single layer consists of a plane of metal atoms sandwiched between two layers of chalcogen atoms. The intralayer M–X bonds are covalent, while adjacent layers of TMDs (in bulk form) are weakly bonded to each other by Van der Walls interactions forces, enabling exfoliation into single layers [48]. Depending on the specific chemical composition, 2D-TMDCs can exist in two different structural phases, the octahedral (1T) and trigonal prismatic (2H) ones (Figure 1d–f). In the notation 2H and 1T, the number indicates the X-M-X units (i.e., the number of layers in the unit cell) while the letter denotes the hexagonal (H) and trigonal (T) symmetries [30,48,49]. According to the chemical composition and depending on the progressive filling of the orbitals, TMDCs can have different opto-electronic behaviour: semiconductors (i.e., MoS_2_, WS_2_), metals (i.e., NbS_2_, VSe_2_), semimetals (i.e., WTe_2_, TiSe_2_) and insulators (i.e., HfS_2_) [30,48,49]. In particular, the TMDCs formed by group-VI transition metals (i.e., MoS_2_, MoSe_2_, WS_2_ and WSe_2_) are widely used for electronic and opto-electronic devices thanks to their charge carrier mobilities (up to 500 cm^2^ V^−1^ s^−1^ in WSe_2_) [17] and bandgaps (ranging from the near-infrared to the visible electromagnetic spectrum) [50,51,52]. Finally, another interesting group of 2D materials is represented by MXenes. Their 3D precursors are characterised by the chemical formula M_n+1_AX_n_ (where M is a transition metal, A is mainly an element of the 13 or 14 group of the periodic table, X is a C and/or N atom, n+1 and n are the numbers of M and X layers, respectively, with n = 1–3); the associated structure is reported in Figure 1g. The 2D form of these materials are produced by etching the A layers [53,54]. After the etching process, the A layers are replaced by surface terminations (indicated with T_x_) such as OH, O and F and so MXenes with chemical formula M_n+1_X_n_T_x_ are synthesized [53,54]. Interestingly, MXenes offer the possibility to tune their optoelectronic properties by choosing properly their chemical composition. For example, their functionalization causes variations of the electrostatic potential near the surfaces, affecting the electronic structure and work function (WF) from 1.6 eV (for OH termination) to 6.25 eV (for O termination) [29,34,55]. These features make MXenes very interesting for the production of SCs; as demonstrated by the Ti_3_C_2_T_x_,the most used material within this family, showing exceptional properties such as high electronic conductivity (6500 S cm^−1^), modest mobility (0.9 cm^2^ V^−1^ s^−1^), and high charge carrier density (3.1∙10^22^ cm^−3^) [56].

## 3. Effects of 2D Materials on Hot Carriers

In PV devices, charge carriers are produced after absorption of photons with energies equal to or far above the bandgap of the light harvester. In the latter case, the photo-generated electrons and holes will populate states above the conduction band minimum (CBM) and below the valence band maximum (VBM), respectively, which are referred to as hot carriers (HCs) [57]. If such HCs can be collected before bringing themselves to thermal equilibrium with the lattice, it is possible to push the PCE of a SC to ~66% (exceeding the Shockley–Quiesser limit of 33% for a single junction SC) [57]. The main issue for the practical realization of HCs based SCs is that HCs relax in an ultrafast timescale, losing the excess energy through two mechanisms: thermalization and cooling [58]. The thermalization process, which occurs within 100 fs, consists in the exchange of energy between carrier–carrier scattering until the HCs reach an equilibrium condition (according to the Fermi–Dirac distribution) with a characteristic temperature *T_C_* [58,59]. In the cooling process, which occurs within 1 ps, the HCs reach thermal equilibrium with the lattice via carrier–phonon scattering [58]. It is worth emphasizing that the HC cooling is one of the main causes for the PCE loss in a SCs (for example ~25% for a MAPbI_3_-based PSCs) [58]. Interestingly, perovskites emerged as promising absorbing material for the realization of HC-based SCs because of the higher HC cooling lifetime (<6 ps for MAPbI_3_, 37 ps for CsPbIBr_2_, 71 ps for MAPbBr_3_ and 305 ps for FAPbI_3_) [60] with respect to other semiconductors (1.4 ps for InN and 2 ps for GaAs) [61,62]. However, it should be mentioned that in addition to large HC cooling lifetimes, energy-selective contacts are necessary for the extraction of the HCs [59]. These energy-selective contacts act as energy filters extracting only carriers with a narrow range of energies and preventing cold carriers (from the external contacts) from cooling HCs [59,63]. In the last few years, 2D materials and particularly graphene rose as potential candidate for the production of energy-selective contacts for efficient charge carrier collection, including HCs [64,65,66].

**Figure 2:**
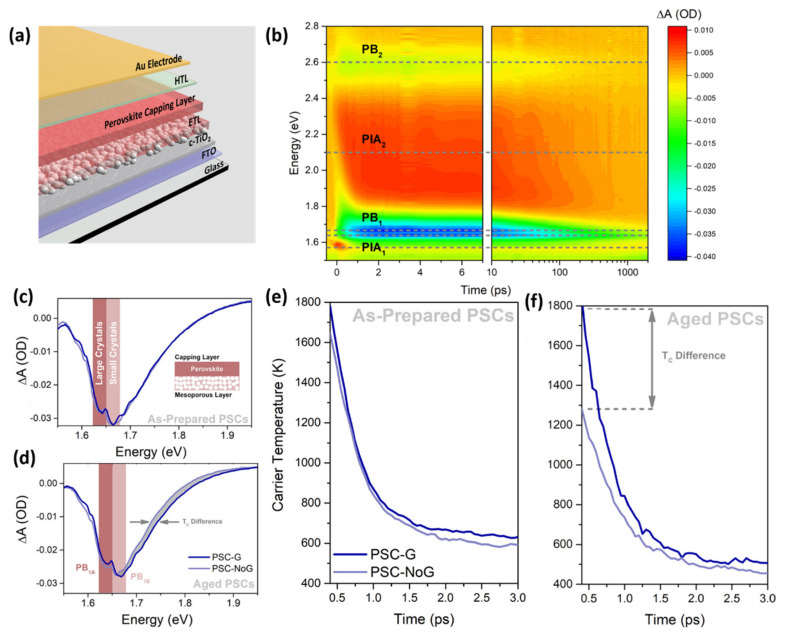
(**a**) Schematic illustration of the architecture of the investigated PSCs. (**b**) Two-dimensional false color map of transient absorption signal of the as-prepared PSC-G. Comparison of the transient absorption signal in the PB_1_ region for (**c**) as-prepared and (**d**) one-week aged PSC-G and PSC-NoG. Comparison of the carrier temperature as a function of time delay for (**e**) as-prepared and (**f**) one-week aged PSC-G and PSC-NoG. Figures adapted from ref. [64]. Copyright American Chemical Society@2019.

For example, O’Keeffe et al. [64] demonstrated that the incorporation of graphene flakes in the m-TiO_2_ based ETL of a PSC is advantageous for the extraction of the HCs allowing the stability and the PCE of mesoporous-based devices to be improved (Figure 2a shows the whole investigated architecture).

In particular, the authors used transient absorption spectroscopy (TAS) to study the dynamics of the carriers within PSCs before and after 1 week of aging. Figure 2b displays the false colour map of the TA spectra as a function of the probe energy and pump-probe time delay for the as-prepared graphene-based PSC (PSC-G). The TA map presents two positive regions ascribed to the photoinduced absorption (PIA_1_ and PIA_2_) of MAPbI_3_ and two negative regions attributed to photobleaching (PB_1_ and PB_2_). Figure 2c shows the comparison of the TAS characterization, in the PB_1_ region, performed on as-prepared PSC-G and reference sample (i.e., without any graphene—PSC-NoG); while Figure 2d reports the spectra measured after aging of the same samples. The focus on the PB_1_ contribution is due to the fact that it represents the only one providing information about the occurring physico-chemical processes (indeed the other regions show only slight variations of the optical density). A more detailed analysis of Figure 2c,d reveals that there are two distinct peaks in the PB_1_ region, which the authors assigned to the absorption bleaching of large perovskite crystals (of the absorbing layer, PB_1A_ centred at 1.64 eV) and in small perovskite crystals (infiltrated within the m-TiO_2_ layer, PB_1B_ at 1.66 eV). Moreover, it is worth noting that the relative intensities of the PB_1A_ and PB_1B_ peaks of the PSC-G sample are the same for the as-prepared device and after aging. By contrast, for the aged PSC-NoG an evident reduction of the relative intensities of PB_1A_ and PB_1B_ is observed with respect to the as-prepared sample. According to the authors, these results suggest that the use of graphene in the m-TiO_2_ layer hinders the degradation of small crystals by avoiding the iodine diffusion from the perovskite in the ETL. Furthermore, as observed in Figure 2d, the photobleaching signal of the PSC-G is wider compared to that of the PSC-NoG. In order to explain this behaviour, it should be mentioned that the shape of the high-energy tail (of Figure 2c,d) is linked to the *T_C_*. In particular, *T_C_* values can be obtained from the TA spectra by fitting the high energy tails with a Maxwell–Boltzman distribution. The *T_C_* as functions of the time delay for the as-prepared and aged PSC-NoG and PSC-G samples are shown in Figure 2e,f. Figure 2e reveals a two-component exponential decay of *T_C_* for both as-prepared PSC-NoG and PSC-G, characterised by a comparable starting *T_C_* (1650 and 1800 K, respectively) and decay lifetimes (the authors obtained, for both samples, values of ~400 fs and 15–20 ps for the two exponential components). Thus, the use of graphene gives only slight variations to the starting HC temperature and decay lifetimes, for the as-prepared samples. On the contrary, Figure 2f shows that PSC-NoG and PSC-G exhibit different starting *T_C_* (1300 K and ~1800 K, respectively) after 1 week of aging but similar two-components decay dynamics with respect to the as-prepared counterparts (~480 fs and 23 ps for the PSC-NoG case, ~420 fs and 15 ps for the PSC-G case). For this reason, the authors propose that graphene acts as a shield for the starting *T_C_* preserving small perovskite crystals (in the m-TiO_2_ layer) from degradation, while in the absence of graphene this contribution is lost (thus charge-carriers suffer from a faster thermalization).

**Figure 3:**
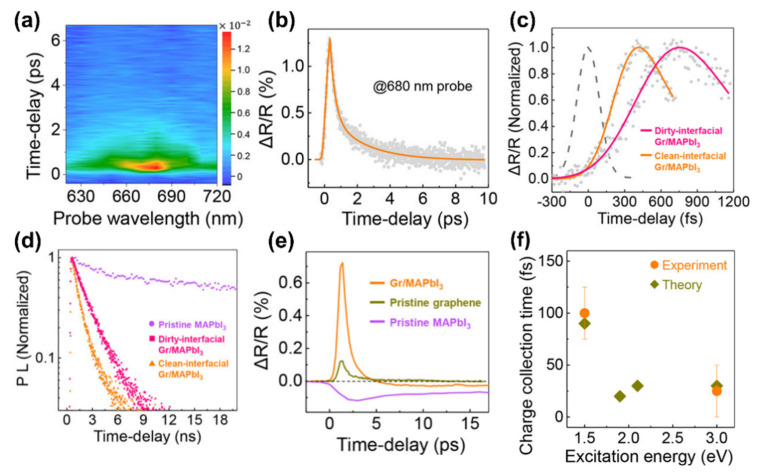
(**a**) Pseudo-colour transient absorption plot of clean graphene/MAPbI_3_ interface at different probe wavelength and with pump wavelength of 820 nm. (**b**) Differential reflection measured from the clean graphene MAPbI_3_ heterostructure with 680 nm probe pulse. (**c**) Normalized differential reflection measured from both dirty- and clean-graphene/MAPbI_3_ interface. The dashed line is the laser cross-correlation function used for the deconvolution of the rising part of the transient absorption signal. (**d**) Normalized time resolved photoluminescence spectroscopy measurements performed on pristine MAPbI_3_ and on clean and dirty graphene/MAPbI_3_ interfaces. (**e**) Differential reflection measured from the pristine MAPbI_3_, the pristine graphene and the clean graphene/MAPbI_3_ interface. (**f**) Comparison of the theoretical time-dependent density function calculations and experimental results of charge collection time of the carriers. Data taken from ref. [65]. Copyright American Chemical Society@2018.

A very interesting study was reported by Hong et al. [65], who performed tunable two-colour pump-probe TAS measurements in combination with theoretical calculations to investigate the HCs dynamics on MAPbI_3_ perovskites grown onto a graphene layer. Interestingly, the authors prepared a *clean* graphene layer through chemical vapour deposition (CVD), without the need of any polymer-based transfer procedure. Afterwards, a very thin (~10 nm) MAPbI_3_ crystal was grown on top of the clean graphene through a one-step solution process (i.e., by putting in contact the graphene surface with a solution of CH_3_NH_3_I and PbI_2_ salts). The 2D map of the TAS signal of the clean graphene/MAPbI_3_ interface, at different probe wavelengths and pump–probe delay times, is shown in Figure 3a, revealing an absorption peak centred at 680 nm.

The vertical cut of the 2D map at 680 nm (Figure 3b) provides the decay of the bleaching signal. Figure 3c shows a restricted range of Figure 3b, together with a measurement performed on a *dirty* graphene/MAPbI_3_ interface (i.e., graphene grown by CVD and transferred by using a polymer). According to these results, the rising part of such spectra indicates a faster charge carrier collection for the clean graphene/MAPbI_3_ interface (~100 fs) with respect to the dirty graphene/MAPbI_3_ one (~530 fs). However, it is fundamental to assess the charge carrier collection efficiency (*η*) to exclude that charge carriers suffer from trapping phenomena in such hetero-structure. For this reason, the authors performed time-resolved photoluminescence spectroscopy measurements on pristine MAPbI_3_ and on the hetero-structures formed with clean and dirty graphene (Figure 3d), obtaining *η* = 98.7% for the clean graphene/MAPbI_3_ case and 97.7% for dirty graphene/MAPbI_3_ sample. Although these results are quite similar, it should be emphasised that the efficient collection of HCs must be realised in the ps time-scale, so slight variations of *η* can make a remarkable difference. Indeed, comparisons of TAS measurements performed on clean graphene, MAPbI_3_ and clean graphene/MAPbI_3_ interface (Figure 3e) reveal the superior HCs collection of the hetero-structure since the peak observed in this characterization is six times higher with respect to the pristine graphene case. Finally, the authors performed time-dependent density functional theory calculations of the charge collection time of carriers at different energies, observing a good agreement with their experimental data (Figure 3f).

**Figure 4:**
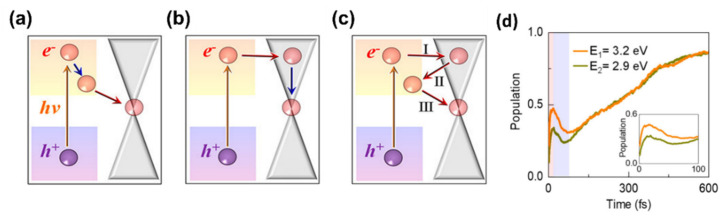
Schematic representation of the photoinduced hot carriers dynamics at MAPbI_3_/graphene interface: (**a**) the hot electrons in MAPbI_3_ relax to the conduction band minimum (CBM) and then transfer to graphene (pathway 1), (**b**) the hot electrons in MAPbI_3_ diffuse to graphene and then relax to the Dirac point (pathway 2), (**c**) the hot electrons in MAPbI_3_ follow a zigzag path between MAPbI_3_ and graphene. (**d**) Population at graphene of the hot electrons with energies of 2.9 and 3.2 eV above the Fermi level of the MAPbI_3_/graphene interface as a function of time after optical excitation. Figures adapted from ref. [66]. Copyright American Chemical Society@2021.

In a following work, the same group conducted further studies on an identical heterostructure with the aim to shed light on the HCs dynamics [66]. Figure 4a–c illustrate the schematic representation of the three possible pathways proposed by the authors to describe the HCs dynamics at MAPbI_3_/graphene heterostructure.

In the first case (pathway1, Figure 4a) the hot electrons (HEs) on the MAPbI_3_ layer relax to the CBM and then diffuse to graphene. For the second process (pathway 2, Figure 4b) firstly, the hot electrons transfer from MAPbI_3_ to graphene and then relax to the graphene Dirac point. Finally, there is an additional process (pathway 3, Figure 4c) in which the hot electrons follow a zigzag walk between the two layers (perovskite and graphene) that form the heterostructure. To identify the pathway followed by HCs, the authors performed a direct non adiabatic analysis and studied the dynamics of HEs with energies of 2.9 and 3.2 eV above the Fermi level of the heterostructure. As shown in Figure 4d, firstly the HEs transfer to graphene in less than 20 fs (the population at graphene of excited HEs increases). After that, the HEs travel back to MAPbI_3_ (there is a drop of the charge population on graphene) on a time scale of ~75 fs (a similar behaviour was already observed in graphene/WS_2_ heterostructure) [67]. Finally, the HEs diffuse from MAPbI_3_ to graphene causing an increase of the population on graphene to 86%. These results indicate that the dynamics of the HCs at MAPbI_3_/graphene interface is described by pathway 3. The overall process takes place on a time scale of ~400 fs that is in good agreement with previous experimental observations that predicted an ultrafast charge collection time of ~100 fs at the graphene/MAPbI_3_ interface [65].

The presented results, although very interesting, concern a very particular case: the interface between CVD graphene and a thin (~10 nm) perovskite crystal. However, in practical devices, perovskite layers usually reach a thickness of hundreds or thousands of nm, thus the straightforward translation of these results into a realistic scenario is not trivial. Further research on this field is fundamental to assess the practical implementation of such a strategy for the realization of HC-based PSCs.

## 4. The Role of 2D Materials in the Growth of High Crystalline Quality Perovskite Films

The crystalline and morphological quality of a perovskite film plays a key role for the realization of PV devices with high performance [68]. Specifically, in order to fabricate a PSC with a high PCE and a good stability, it is important to grow a perovskite layer that is uniform and smooth with a low amount of defects (to avoid the presence of a large number of recombination centres and loss of voltage) and large grain size (that favours an efficient transport of charge carriers to the electrodes) [69,70,71,72,73]. With the aim to improve the perovskite film quality, that depends on the preparation method used (such as one or two step solution process, co-evaporation or spin coating) [74,75,76] and other factors (for example the choice of the solvent or the precursor composition and annealing treatment) [77,78,79], several approaches including new deposition technologies [80,81], post-treatment [82] and additive engineering [83,84] have been proposed. In particular, the use of 2D materials within the perovskite precursor solution represents a promising route to control the nucleation and growth processes of perovskite films.

**Figure 5:**
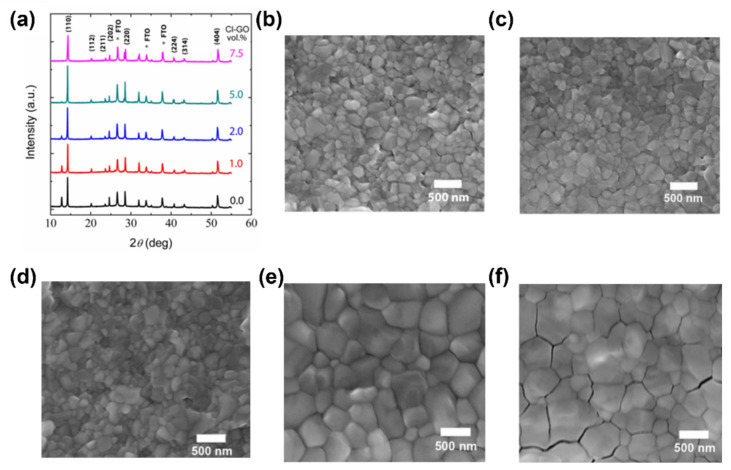
(**a**) XRD patterns of perovskite films as a function of Cl–GO amounts. SEM images of perovskite films with different amounts of Cl–GO in the precursor solution: (**b**) pristine 0.0 vol%, (**c**) 1.0 vol%, (**d**) 2.0 vol%, (**e**) 5.0 vol%, and (**f**) 7.5 vol% Cl-GO. Data taken from ref. [85]. Copyright Springer Science Business Media, LLC, part of Springer Nature@2020.

For instance, Gidey et al. used chlorine functionalized GO (Cl-GO) as additive into a MAPbI_3_ precursor solution, resulting in high quality of the perovskite layer and leading to improved PCE of the device compared to the pristine MAPbI_3_ based PSC [85]. To study the structure and the morphology of MAPbI_3_ with different amounts of Cl-GO (1.0 vol%, 2.0 vol%, 5.0 vol% and 7.5 vol%) the authors performed X-ray diffraction (XRD) and scanning electron microscopy (SEM) measurements and compared the results with that of pristine MAPbI_3_. The XRD results (Figure 5a) show two main characteristic peaks at 14.2° and 28.4°, corresponding to the crystallographic planes (110) and (220). Remarkably, for all the samples there is no shift of the peaks positions, indicating that both pristine MAPbI_3_ and MAPbI_3_-Cl-GO films share the same crystalline structure, however several variations in the shape and intensity of the pattern are observed (underlying different crystallite dimensions, in accordance with Sherrer’s relation). In fact, Figure 5b-f report the SEM images of both pristine MAPbI_3_ and MAPbI_3_ films prepared with different amounts of Cl-GO, confirming that grain size increases with the addition of Cl-GO up to 5.0 vol% concentrations. However, with higher Cl-GO concentration (7.5 vol%) the perovskite film was subjected to the formation of cracks within the grain boundaries that has a negative impact on the performances of the PSC. Indeed, the PCE of the devices fabricated by the authors is boosted from 12.81% to 15.14% by increasing the amount of Cl-GO from 0.0% to 5.0% and decreases to 14.16% in the case of MAPbI_3_-Cl-GO 7.5 vol% based PSC. Thus, these results reveal that the proper addition of Cl-GO has a beneficial impact on the nucleation and crystal growth processes of metal halide perovskites.

**Figure 6:**
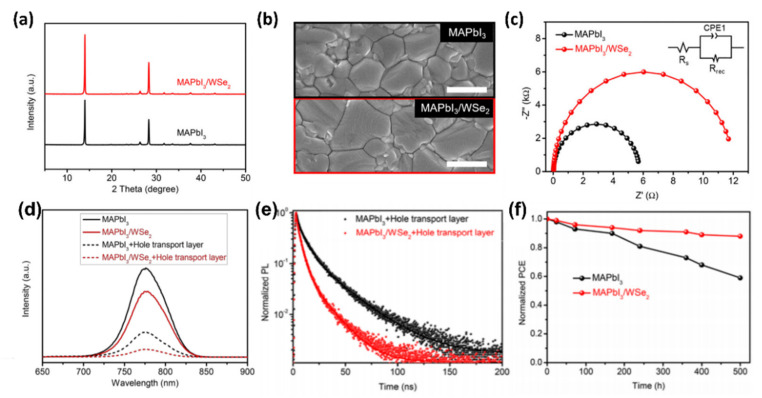
(**a**) XRD patterns of MAPbI_3_ and MAPbI_3_/WSe_2_ films; (**b**) SEM images of MAPbI_3_ and MAPbI_3_/WSe_2_ films; (**c**) Nyquist plots of MAPbI_3_ and MAPbI_3_/WSe_2_-based PSCs; (**d**) PL spectra of the MAPbI_3_ and MAPbI_3_/WSe_2_ films with and without hole-transporting layer (HTL); (**e**) TRPL decay curves of the MAPbI_3_+HTL and MAPbI_3_/WSe_2_+HTL structures; (**f**) Photostability of non-encapsulated MAPbI_3_ and MAPbI_3_/WSe_2_ based PSC devices under storage in N_2_ glovebox (illumination of 100 mW cm^−2^ white light-emitting diode (LED) for 500 h). Data taken from ref. [86]. Copyright American Chemical Society@2021.

Similar results in the improvement of the crystallization process to obtain high-quality perovskite films with uniform morphology and large average grain size were obtained by Qin et al. [86] through the addition of quasi-monolayer WSe_2_ nanosheets into MAPbI_3_ precursor solution. The average thickness of the quasi-monolayer WSe_2_ nanosheets (synthesized by liquid phase exfoliation) is ~0.8 nm (the same thickness values are reported for a monolayer of WSe_2_). Specifically the role of WSe_2_ in the growth of MAPbI_3_ film was investigated by using XRD and SEM techniques (Figure 6a,b).

In particular, as shown in Figure 6a, the MAPbI_3_-WSe_2_ film displays the same XRD features, ascribed to MAPbI_3_ crystals (with the main peaks located at 14.2° and 28.4°) [85]. Furthermore, in both XRD spectra no relevant shift of the position of the peaks is observed, indicating that after the introduction of WSe_2_ into the MAPbI_3_ precursor solution the crystal structure of the perovskite is not altered. Significantly, the relative intensities of the peaks of the MAPbI_3_/WSe_2_ layer are higher compared to those of the pristine MAPbI_3_ film, suggesting an enhanced crystalline quality. Figure 6b presents the SEM images of the MAPbI_3_ and MAPbI_3_/WSe_2_ films, respectively. The average crystal size is larger for the MAPbI_3_/WSe_2_ sample, confirming that WSe_2_ is an effective additive for controlled nucleation and growth of perovskite films. Moreover, to truly understand the charge carrier transport and recombination behaviour, the authors conducted electrochemical impedance spectroscopy (EIS) measurements. The Nyquist plots of the MAPbI_3_ and MAPbI_3_/WSe_2_-based PSCs are depicted in Figure 6c (in the inset the equivalent circuit model is represented). In these plots the semi-circles in the high and low frequency regions are assigned to the recombination resistance (R_rec_) and series resistance (R_S_) respectively. In particular, by fitting the Nyquist plots, R_rec_ values of 12 kΩ and 6 kΩ were obtained for the MAPbI_3_/WSe_2_- and MAPbI_3_-based PSCs, implying that the WSe_2_ nanosheets suppressed the charge carrier recombination. In addition, the charge carrier extraction properties were analysed by both steady state PL and TRPL experiments. As shown in Figure 6d, the PL intensity of the MAPbI_3_/WSe_2_ layer with the HTL deposited on top is lower than that of the other structures examined, resulting in an improved hole extraction ability and confirming the reduced charge carrier recombination induced by the WSe_2_ nanosheets. This result is also supported by the trend of the TRPL decay curves of both MAPbI_3_+HTL and MAPbI_3_/WSe_2_+HTL structure (Figure 6f). Indeed, a faster decay is observed for the MAPbI_3_/WSe_2_+HTL sample underlying again the beneficial role that the WSe_2_ has in the hole transport. Finally, the stability of both MAPbI_3_ and MAPbI_3_-WSe_2_-based PSCs was investigated and the result is shown in Figure 6g. It is clear that the MAPbI_3_/WSe_2_-based PSC retains 91% of its initial PC) after storage in a N_2_ glovebox for 500 h under continuous illumination (100 mW cm^−2^ white LED), while the reference MAPbI_3_ based PSC suffers from severe degradation with a PCE retention of 60% under the same aging conditions. The enhanced stability of the MAPbI_3_/WSe_2_-based PSC is attributed by the authors to the passivation of Pb^0^ defect due to the formation of Pb-Se coordination bonds between WSe_2_ and MAPbI_3_.

## 5. The Role of 2D Materials for Electronic Energetics Tuning and Interface Engineering in Perovskite Solar Cells

**Figure 7:**
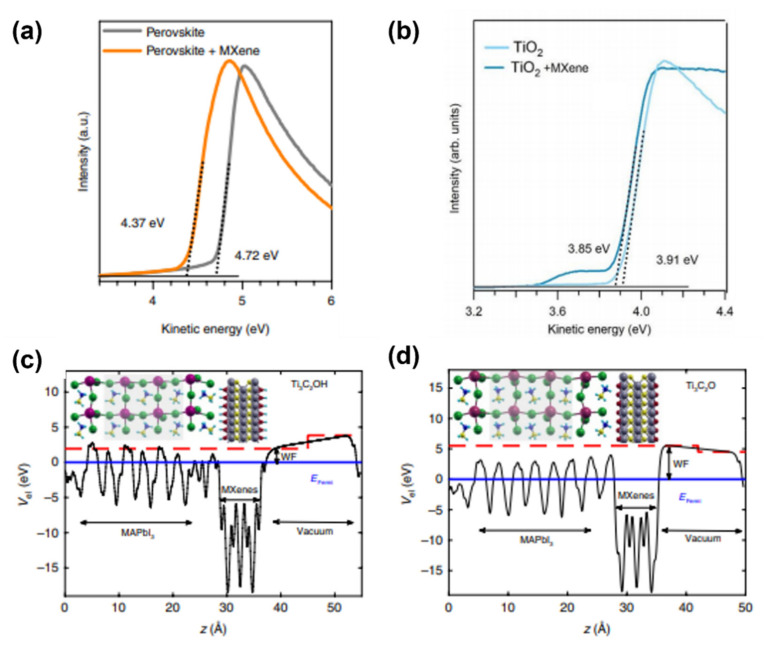
UPS spectra of (**a**) MAPbI_3_ and (**b**) TiO_2_ doped with Mxenes. Results of DFT calculations concerning the electrostatic potential of planes perpendicular to the (**c**) MAPbI_3_/Ti_3_C_2_(OH)_2_ and (**d**) MAPbI_3_/Ti_3_C_2_O_2_ interface. Data taken from ref. [90]. Copyright Springer Nature@2019.

In a PSC, the absorbing layer is sandwiched between the ETL and the HTL, and thus one of the most important prerequisites to achieve high performance is the optimal alignment of the energy levels at the interfaces. Since 2D materials exhibit a tunable electronic structure by varying their chemical composition or functionalization, they hold great promises for engineering such interfaces. In this regard, Agresti et al. used Ti_3_C_2_T_x_ as an additive within a triple cation perovskite and TiO_2_, with the aim to tune the corresponding WFs. Ultraviolet photoemission spectroscopy (UPS) measurements (Figure 7a,b) reveal a shift of WF from 4.72 to 4.37 eV (for perovskites) and from 3.91 to 3.85 eV (for TiO_2_). In the case of the doped-TiO_2_, the slight WF variation is due to the partial oxidation of MXene during the annealing necessary for the TiO_2_ sintering process. To shed light on the mechanisms underlying the WF changes in the doped-triple cation perovskite, the authors performed density functional theory (DFT) calculations of a doped-MAPbI_3_ structure considering OH and O terminations. Calculations were performed by using MAPbI_3_ to make the computation feasible and because the same WF shift was also observed for such perovskite. The results are shown in Figure 7c,d that reveal the formation of a dipole at the MAPbI_3_/Ti_3_C_2_T_x_ interface, thus the WF tuning arises from electrostatic effects. The same result was obtained for simulated doped-FAPbI_3_, while for the case of doped- CsPbI_3_ the WF is expected to be higher because of the larger band gap of Cs-based perovskites. Thus, for the triple cation perovskite used by the authors, an overall reduction of the WF is expected (since Cs constitutes only the 5% of the overall inorganic cation concentration).

Due to the possibility to play with MXenes terminations during their chemical synthesis, theoretically the whole phase-space of F, OH, and/or O mixtures [87] could be explored in combination with different perovskite compositions, opening the way to a general approach for engineering optoelectronic devices based on a perovskite active layer. Moreover, the proposed strategy is suitable for different PSC structures. Indeed, when applied in mesoscopic n-i-p architecture, the reduction of perovskite WF mediated by the Ti_3_C_2_T_x_ MXenes was effective for reducing the valence band barrier at the perovskite/spiro-OMeTAD interface, leading to an increased open circuit voltage (V_OC_). A similar engineering strategy was recently reported for inverted p-i-n architecture where a MXene-doped MAPbI_3_ absorber was grown over a NiO_x_ HTL and topped with a PCBM/BCP/Ag negative electrode [88]. Notably the same MXenes precursor was here used to dope both perovskite and PCBM layers, in order to engineer the energy level alignment at MAPbI_3_/PCBM interface while improving the charge dynamics within the whole device structure. Indeed, a Ti_3_C_2_T_x_ with the ratio ~1 between ‒OH to ‒O terminations was selected for achieving 4.35 eV WF value, suitable for inducing a negative shift in both MAPbI_3_ and PCBM materials, responsible for a remarkable enhancement in the photocurrent generation, considering the red-NIR part of the visible spectrum. The as-modified inverted PSC achieved efficiency above 19%, a remarkable result when considering the standard p-i-n architecture based on NiO_x_ HTL and MAPbI_3_ without adding complexity to the cell fabrication process as in the case of additional interlayers or passivation steps. As a matter of fact, the interface engineering based on MXenes was demonstrated as an effective tool in boosting the performance of PSC employing a fully inorganic CsPbBr_3_ perovskite active layer and carbon electrode, aiming for a long lifetime under real working condition [89]. In particular, Ti_3_C_2_-MXenes were inserted as an interlayer between perovskite and carbon top electrode (FTO/TiO_2_/CsPbBr_3_/Ti_3_C_2_/carbon) for eliminating the energy level mismatch, by accelerating the hole extraction and reducing the recombination at the interface. Moreover, the nanosheets MXene functional groups such as ‒O also provide strong interactions between the MXenes and under-coordinated Pb atoms, which led to a remarkable reduction of the deep trap defect concentration in the CsPbBr_3_ films. Notably, the MXene-engineered carbon-based cell demonstrated long-term stability for over 1900 h in a moisture environment and more than 600 h under thermal conditions, by ensuring PCE above 9%.

## 6. Promises and Challenges for the Large-Scale Use of 2D Materials in PSCs

With these premises, the interface engineering strategy based on 2D materials could assume a pivotal role in the future in pushing the development of commercialization of perovskite-based solar cells, still hampered by the not unequivocally demonstrated device stability and difficult device scalability. Indeed, as main concern, the astonishing results in terms of PCE demonstrated over lab-scale devices are still far from being equalled by large-area modules, while stability under established stress test protocols [91] was not still fully addressed. To this extent, Agresti et al. [92] recently reported a valuable example of interface engineering strategy based on 2D materials and applied to a perovskite large area module [93,94]. More in detail, the easy tuning of 2D material optoelectronic properties is here exploited for shifting the MoS_2_ valence band edge that does not perfectly match with the perovskite highest occupied molecular orbital (HOMO) level and possibly forms an energy barrier for the hole extraction process. The MoS_2_ energy bands up-shift was obtained by linking the thiol group of 3-mercaptopropionic acid moieties to the MoS_2_ surface via S−S van der Waals physisorption and/or S-vacancy passivation. Once engineered in terms of energy band, the as-functionalized MoS_2_ (fMoS_2_) was inserted as interlayer at perovskite/HTL interface with the double role to improve the hole extraction process (by reducing the potential barrier at the perovskite/HTL interface), while restricting the undesired electron transfer from perovskite (since the conduction band edge of MoS_2_ above the LUMO level of the perovskite proves electron blocking properties). When fMoS_2_ was inserted as interlayer in a device structure employing even graphene-modified photo-electrode (FTO/cTiO_2_+G/mTiO_2_+G/perovskite/fMoS_2_/spiro-OMeTAD/Au), modules with 13.4% PCE were demonstrated with an active area larger than 108 cm^2^. Here, the rational use of 2D materials allows the PCE efficiency gap usually experienced when moving from small to large area devices to be reduced, by acting at the perovskite/charge transport layer interfaces where charge recombination has even more negative impact on the performance of the device, the larger the interface surfaces. As a feasible perspective, 2D material-based engineering, together with the use of a carbon/graphene-based electrode replacing the gold one [95], may finally ensure a way to (i) scale-up the perovskite-based devices without losses in PCE; (ii) keep the fabrication cost as low as possible thanks to the possibility to produce 2D materials in an easy, cheap and scalable way through solution processes, while using a roll2roll or sheet2sheet high-throughput production [96] thanks to the fully printable carbon-based device structure. Finally, it is worth mentioning that perovskites themselves show 2D phases, which are obtained by inserting hydrophobic cations within the perovskite structure [7]. This results in a higher environmental stability but in very different optoelectronic properties with respect to the 3D case. In particular, 2D perovskites have higher band-gaps and exciton binding energies (arising from quantum confinement effects) [7,97], thus SCs based on pure 2D perovskites usually exhibit lower PCEs, although further advancements are leading to very good improvements (for examples, some works have reported PCEs up to 18.5%) [98,99]. Quite differently, the combination of the high efficiency of 3D perovskites and the high stability of 2D perovskites, within the same SC, has been demonstrated as a successful strategy to guarantee long lifetime devices [100], making PSC technology competitive with Si-based PVs. We encourage the interested readers to consult the following reviews for a deeper understanding of this very interesting field of perovskite technologies [7,101,102,103].

## 7. Conclusions

Nowadays, perovskite solar cells (PSCs) have emerged as ideal candidates for solar energy conversion owing to their outstanding power conversion efficiency (PCE) of 25.5% and the possibility of fabrication through low-cost processes. Several strategies have been proposed to further enhance the PCEs of these devices, such as interface engineering, chemical functionalization, etc. In this context, two-dimensional (2D) materials including graphene and its derivatives, transition metal dichalcogenides (TMDCs such as MoS_2_, WS_2_), transition metal carbides, nitrides and carbonitrides (MXene, such as Ti_3_C_2_T_x_) have attracted great interest due to their remarkable opto-electronic properties, chemical tuning and stability. This review provides a brief overview of some effects induced by the addition of 2D materials within PSCs. Firstly, it we emphasized the role of 2D materials for the efficient collection of hot carriers, paving the way for hot-carrier based solar cells (with a maximum theoretical PCE of ~66%). Moreover, when used as additives in the perovskite precursor solution, 2D materials help the formation of a perovskite layer with elevated crystalline quality (larger grain size). Finally, it was shown how 2D materials can be used to tune the work function of perovskites and charge-extracting layers thank to the formation of a dipole moment at the interface between such components. Thus, the exploitation of 2D materials represents a promising way to realize highly efficient and low-cost PSCs, and a feasible strategy to definitively push their commercialization through easy and cheap high-throughput production processes.

## Figures and Tables

**Table 1:** Band gap and carrier mobility of some representative 2D materials.

Material	Band Gap (eV)	Carrier Mobility (cm^2^V^−1^s^−1^)	Ref
Graphene	0	~2 × 10^5^	[17]
MoS_2_	1.2–1.8	10–200	[17]
WS_2_	1.3–2.1	43–234	[18,19,20]
WSe_2_	1.2–1.7	140–500	[17]
SnS_2_	2.18–2.44	50–230	[21,22]
TiS_2_	0.02–2.5	7.24	[23,24]
Ti_2_CO_2_	0.91	~400	[25,26]
HfCO_2_	1.79	~1100	[25,27]
Zr_2_CO_2_	1.76	~600	[25,27]

## Data Availability

Not applicable.
